# Graphene-Based Nanoresonator with Applications in Optical Transistor and Mass Sensing

**DOI:** 10.3390/s140916740

**Published:** 2014-09-09

**Authors:** Hua-Jun Chen, Ka-Di Zhu

**Affiliations:** Key Laboratory of Artificial Structures and Quantum Control (Ministry of Education), Department of Physics and Astronomy, Shanghai Jiao Tong University, Shanghai 200240, China

**Keywords:** graphene nanoresonator, optical transistor, nonlinear optical spectroscopy, mass sensing

## Abstract

Graphene has received significant attention due to its excellent properties currently. In this work, a nano-optomechanical system based on a doubly-clamped Z-shaped graphene nanoribbon (GNR) with an optical pump-probe scheme is proposed. We theoretically demonstrate the phenomenon of phonon-induced transparency and show an optical transistor in the system. In addition, the significantly enhanced nonlinear effect of the probe laser is also investigated, and we further put forward a nonlinear optical mass sensing that may be immune to detection noises. Molecules, such as NH_3_ and NO_2_, can be identified via using the nonlinear optical spectroscopy, which may be applied to environmental pollutant monitoring and trace chemical detection.

## 1. Introduction

Graphene, the excellent two-dimensional (2D) material, has drawn tremendous attention in recent years. Due to its unique properties of low mass density, high frequency, high quality-factor and intrinsically small size, graphene is considered as an ideal material for fabricating nanomechanical systems (NMSs) [1–4]. As a result, graphene mechanical resonators have potential applications in force detection [5–7] and mass sensing [8,9]. Currently, by virtues of low mass and stiffness, graphene-based optomechanical systems have been demonstrated experimentally [10–13].

Recently, graphene-based transistore have also been achieved experimentally [14–18]. Nevertheless, although graphene behaved as semimetal, monolayer graphene is a zero-bandgap nanomaterial, and a few applications requiring a bandgap will be limited due to this intrinsic property. Quasi-one-dimensional graphene nanoribbon (GNR) can exhibit a bandgap via quantum confinement, which has been demonstrated recently [19,20], and a number of various experimental techniques have been developed to fabricate GNRs [20–22]. There has been substantial progress towards the goal of controlling the GNRs edge termination with armchairs and zigzag edge geometries at present [23,24]. Since GNRs have a tunable bandgap sensitive to the size and geometry, they are good candidates for possible electronic and optical devices. Here, we will firstly present an optical transistor based on the GNR-optomechanical system (concrete descriptions are given later in the Results and Discussions).

Besides, graphene also owns the property of a high surface-to-volume ratio, which is a natural asset for applications in sensing [25–27]. Additionally, electromechanical measurements with graphene resonators suggest that graphene-based NMSs devices have the potential to be fabricated as ultrasensitive mass sensors [1]. Resonating nanomechanical resonators can be used for mass sensing, because the deposited mass on the surface of the resonator will result in a change of the resonance frequency [1,9,28]. Typical mass spectrometry mostly concentrates on electrical circuitry or the optical read-out scheme. In electrical measurement schemes, mechanical signals are transformed into electric signals to realize the frequency measurement. However, the heat effect and energy loss caused by the electric circuitry will broaden the electrical response spectrum and finally affect the sensitivity of the frequency detection [29,30]. In the optical read-out scheme, a laser beam is focused onto the free end of the resonator, and the reflection is monitored using a position-sensitive photodiode; while fabrication of the devices for piezoresistive read-out is more time-consuming. Recently, nanomechanical resonator-based mass sensors with the optical pump-probe scheme have been proposed in the all-optical domain [31–33]. However, the proposal still focuses on the linear optics regime, and the mass sensing schemes with nonlinear optics have received little attention until now. Here, based on the GNR-optomechanical system, we propose nonlinear optical mass sensing. Compared with the mass sensing in the linear optics regime [33], the nonlinear optical mass sensing proposed here may be immune to detection noises (concrete elaborations are given later in the Discussions).

In this present article, we theoretically study and analyze the coherent optical properties of a graphene resonator based on a doubly-clamped Z-shaped GNR [33,34] with the optical pump-probe scheme [35,36]. We investigate the phenomenon of phonon-induced transparency (PIT) in the resonator system. Electromagnetically-induced absorption (EIA) and parametric amplification (PA) are also demonstrated, with controlling the pump field intensity, which may serve as a quantum optical transistor. Further, the enhanced optical Kerr effect can also be modulated instantaneously via tuning the intensity of the pump field, and we further propose a nonlinear optical mass sensing with the optical Kerr effect based on the GNR resonator system. By measuring the resonance frequency shift of the GNR resonator of the nonlinear optical spectroscopy, the mass of external particles deposited on the GNR can be determined distinctly. The scheme proposed here may have potential applications in mass sensing and all-optical graphene-based devices.

## 2. Model and Theory

**Figure 1.**
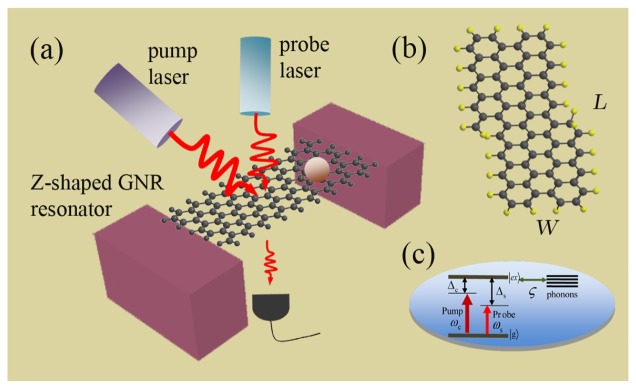
**(a)** Schematic diagram of a doubly-clamped Z-shaped graphene nanoribbon (GNR) resonator system with the optical pump-probe scheme; **(b)** Atomic structure of the Z-shaped GNR; **(c)** The exciton energy levels in the Z-shaped GNR coupled to the resonator. If NH_3_ and NO_2_ molecules are deposited onto the surface of the resonator, their mass can be determined immediately by the frequency shift of the nonlinear optical spectroscopy.

The model consisted of a doubly-clamped Z-shaped GNR resonator, as shown in Figure 1a. Due to the special band structure, graphene's low-energy quasi-particles will behave as Dirac fermions, and the Klein tunneling and chiral effect make it nontrivial to form good quantum dots (localized electron states) in graphene [37]. Several methods have been conceived of to localize electrons (holes) in graphene at present [34,38–41]. GNRs have the form of armchair GNRs [23] and zigzag GNRs [34], which depend on the cutting direction. Scanning tunneling spectroscopy shows that zigzag and armchair edges exhibit different features of the standing wave patterns [42]. In the single-mode regime, zigzag GNRs show perfect conductance for limited scattering to long-range impurities only, which is attributed to the single-valley transport induced by the existence of a chiral mode propagating at the edge of the zigzag GNRs. Moreover, a strain along graphene's zigzag direction might eventually lead to a gap opening at large deformations. Due to these properties, we consider a Z-shaped GNR as a nanomechanical resonator. We propose an alternative approach in which the localized states can exist in the zigzag region of a Z-shaped GNR (see Figure 1b), and its edge bonds are saturated by ordinary hydrogen atoms. As the Z-shaped junction device can completely confine electronic states induced by the topological structure of the junction and by varying the length of the junction, the spatial confinement and the number of discrete levels are modified accordingly [34,41]. A two-level system with the ground state |g〉 and the first excited state (single exciton) |*ex*〉 characterized by the pseudospin – 1/2 operator *S^z^* (*S*^±^) is introduced to describe the exciton in the Z-shaped GNR [33], and the energy level is shown in Figure 1c. Therefore, the Hamiltonian of this localized two-level exciton can be described by *H_e_* = *ħω_e_S^z^* with the frequency *ω_e_* of the exciton.

Clamping and suspending the Z-shaped GNR with SiO_2_ substrate (see Figure 1a), a doubly-clamped suspended Z-shaped GNR nanomechanical resonator is constructed [33,43]. In the resonator system, we introduce a resonator-bath representation, with the resonator mode (bosonic annihilation and creation operators Φ and Φ^+^) corresponding to the fundamental in-plane flexural resonance and the bath, including the other GNR vibrational resonances coupled to the 3D substrate that supports the Z-shaped GNR [43]. The lowest-energy resonance corresponds to the fundamental flexural mode, and the resonator is assumed to be characterized by sufficiently high quality factors *Q*. The eigenmode is described by a quantum harmonic oscillator, and the Hamiltonian is *H_r_* = *ħω_m_*(Φ^+^Φ + 1/2), with the resonator frequency *ω_m_*. The excitonic resonance in the GNR resonator plays the same role of the optical cavity that couples to the flexural motion via deformation potential electron-phonon interactions. Then, the interaction between the resonator and exciton is *H_r_*_−_*_e_* = *ħω_m_ζS^z^* (Φ^+^ + Φ), and ζ is the coupling strength [33,43].

Applying a strong pump laser (with frequency *ω_c_*) and a weak probe laser (with frequency *ω_s_*), termed optical pump-probe technology [35,36], to the GNR resonator system, we obtain the whole Hamiltonian [44] of the coupled system as:

(1) $$H=H_{e}+H_{r}+H_{r-e}-\mu \sum_{k=c,s}E_{k}(S^{+}e^{-i\omega_{k}t}+S^{-}e^{i\omega_{k}t})$$

where *μ* is the electric dipole moment of the exciton in the GNR resonator and *E_c_* (*E_s_*) is the amplitude of the pump (probe) field. The dynamics of the coupled Z-shaped GNR resonator system in the presence of dissipation and dephasing is described by the following master equation [45]:

(2) $$\dot{\rho }=-i[H,\rho ]/\hbar +\Gamma_{1}L[S^{-}]\rho /2+\gamma_{m}L[\Phi ]\rho +\gamma P[S^{z}]\rho /2$$

where *ρ* is the density matrix of the coupled system, Γ_1_ is the exciton relaxation rate, *γ* is the pure dephasing rate of the exciton and *γ_m_* is the decay rate of the GNR resonator. 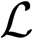
[*D*]*ρ* = 2*DρD*^+^ −{*D*^+^*D*, *ρ*} and 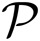
[*D*]*ρ* = *DρD*^+^ − *ρ* are the Lindblad operator describing the incoherent decays. Using the identity 〈*Ȯ*〉 = *Tr*(*Oρ)* for an operator *O* and a density matrix *ρ* in the above equation, in the rotating frame at the pump laser frequency *ω_c_*, we obtain the following Bloch equations for the coupled GNR resonator system as:

(3) $$\langle {\dot{S}}^{z}\rangle =-\Gamma_{1}\left(\langle S^{z}\rangle +1/2\right)+i\Omega_{c}\left(\langle S^{+}\rangle -\langle S^{-}\rangle \right)+(i\mu E_{s}/\hbar )\left(\langle S^{+}\rangle e^{-i\delta t}-\langle S^{-}\rangle e^{i\delta t}\right),$$

(4) $$\langle {\dot{S}}^{-}\rangle =-\left[i\left(\Delta_{c}+\omega_{m}\varsigma \langle \Psi \rangle \right)+\Gamma_{2}\right]\langle S^{-}\rangle -2i\Omega_{c}\langle S^{z}\rangle -2i\mu E_{s}e^{-i\delta t}\langle S^{z}\rangle /\hbar ,$$

(5) $$\langle \ddot{\Psi }\rangle +\gamma_{m}\langle \dot{\Psi }\rangle +\omega_{m}^{2}\langle \Psi \rangle =-2\omega_{m}^{2}\varsigma \langle S^{z}\rangle$$

where the position operator Ψ = Φ^+^ + Φ, Δ*_c_* = *ω_ex_* − *ω_c_* is the detuning of the exciton frequency and the pump frequency, Ω*_c_* = *μE_c_*/*ħ* is the Rabi frequency of the pump field and *δ* = *ω_s_* − *ω_c_* is the probe-pump detuning. Γ_2_ is the dephasing rate of the exciton satisfying Γ_2_ = (Γ_1_ + *γ*)/2. If the pure dephasing rate is neglected (*γ* = 0), then Γ_1_ = 2Γ_2_.

In order to solve these equations, we first take the semiclassical approach by factorizing the GNR resonator and exciton degrees of freedom (〈Ψ*S*^−^〉 ≈ 〈Ψ〉 〈*S*^−^〉), where any entanglement between these systems should be ignored [46]. Here, we make the ansatz [47]:

(6) $$\langle O\rangle =O_{0}+O_{+}e^{-i\delta t}+O_{-}e^{i\delta t},O=S^{z},S^{-},\Psi$$

Inserting these operators into Equations (3)–(5) and neglecting the nonlinear terms, solving the equation set and working to the lowest order in *E_s_*, but to all orders in *E_c_*, the linear optical susceptibility can be derived as $\chi_{\text{eff}}^{\left(1\right)}(\omega_{s})=\mu S_{+}(\omega_{s})/E_{s}=\sum_{1}\chi^{\left(1\right)}(\omega_{s})$, where Σ_1_ = *μ*^2^/(*ħ*Γ_2_) and *χ*^(1)^(*ω_s_*) is given by:

(7) $$\chi^{\left(1\right)}(\omega_{s})=\frac{\left[(\Lambda_{4}^{*}+\varrho_{1}^{*}\Lambda_{3})\Lambda_{1}\varrho_{2}-in_{0}\Lambda_{4}^{*}\right]\Gamma_{2}}{\varrho_{1}^{2}\Lambda_{1}\Lambda_{3}^{*}+\Lambda_{2}\Lambda_{4}^{*}}$$

where *ϱ*_1_ = *i*Ω*_c_*/(Γ_1_ − *iδ*), $\varrho_{2}=iS_{0}^{-*}/(\Gamma_{1}-i\delta )$, *ϱ*_3_ = *i*Ω*_c_*/(Γ_1_ + *iδ*), $\varrho_{4}=iS_{0}^{-}/(\Gamma_{1}+i\delta )$, $\nu =-2\omega_{m}^{2}\varsigma /(\omega_{m}^{2}-\delta^{2}-i\delta \gamma_{m})$, $\Lambda_{1}=-i(2\Omega_{c}+\omega_{m}\varsigma S_{0}^{-}\nu )$, Λ_2_ = *i*(Δ*_c_* + *ω_m_*ζΨ_0_ − *δ*) + (Γ_2_ + Λ_1_*ϱ*_1_), $\Lambda_{3}=-i(2\Omega_{c}+\omega_{m}\varsigma S_{0}^{-}\nu *)$, Λ_4_ = *i*(Δ*_c_* + *ω_m_*ζΨ_0_ + *δ*) + (Γ_2_ + Λ_3_*ϱ*_3_) (Ξ* indicates the conjugate of Ξ). The population inversion $\left(n_{0}=2S_{0}^{z}\right)$ of the exciton related to Ψ_0_ and $S_{0}^{-}$ is determined by:

(8) $$\Gamma_{1}(n_{0}+1)[\Gamma_{2}^{2}+{\left(\Delta_{c}-\varsigma^{2}\omega_{m}n_{0}\right)}^{2}]+4\Gamma_{2}\Omega_{c}^{2}n_{0}=0$$

The imaginary and real parts of *χ*^(1)^(*ω_s_*) indicate absorption and dispersion, respectively. Actually, the above derivations have been obtained in [33]. In order to investigate the nonlinear optical property of the GNR resonator system, we simultaneously derive the nonlinear optical susceptibility as $\chi_{eff}^{\left(3\right)}(\omega_{s})=\mu S_{-}(\omega_{s})/(3E_{c}^{2}E_{s})=\sum_{3}\chi^{\left(3\right)}(\omega_{s})$ with $\sum_{3}=\mu^{4}/\left(3\hbar^{3}\Gamma_{2}^{3}\right)$, and *χ*^(3)^(*ω_s_*) is shown as:

(9) $$\chi^{\left(3\right)}(\omega_{s})=\frac{\left[(\Lambda_{2}^{*}+\Lambda_{3}^{*}\Lambda_{1})\Lambda_{3}\varrho_{4}-iw_{0}\Lambda_{3}\varrho_{3}\right]\Gamma_{2}^{3}}{(\Lambda_{4}\Lambda_{2}^{*}-\varrho_{3}^{2}\Lambda_{3}\Lambda_{1}^{*})\Omega_{c}^{2}}$$

The real and imaginary parts of *χ*^(3)^(*ω_s_*) characterize the Kerr coefficient and nonlinear absorption, respectively.

Based on the nonlinear optical Kerr spectroscopy, a nonlinear optical mass sensing can be implemented. Although the technique of the mass sensor is challenging, the principle of mass sensing is quite simple. Here, the basic principle for mass sensing is mainly measuring the shift of the resonance frequency of the resonator when the external small mass is added on the resonator. Generally, the mass *Sm* to be weighed is deposited on the surface of GNR resonator with mass *M* (*M* ≫ *δm*), whose resonant frequency *ω_m_* will be shifted to *ω_m_* + *δf*. By detecting the frequency shift *δf*, the mass of deposited particle can be weighed as:

(10) $$\delta m={ℜ}^{-1}\delta f$$

where ℜ = *ω_m_*/(2*M*) is the mass responsivity [48]. To deposit additional mass on the surface of the GNR resonator, the gas nozzle aperture can provide a controlled flux of molecules. The flux is gated by a mechanical shutter to provide calibrated, pulsed mass accretions upon the mechanical resonator, and such a technique has been used experimentally [49]. Due to *M* ≫ *δm*, here, we assume that additional mass are distributed uniformly on the resonator, and the added mass does not affect the spring constant of the resonator and the coupling strength.

## 3. Results and Discussions

**Figure 2.**
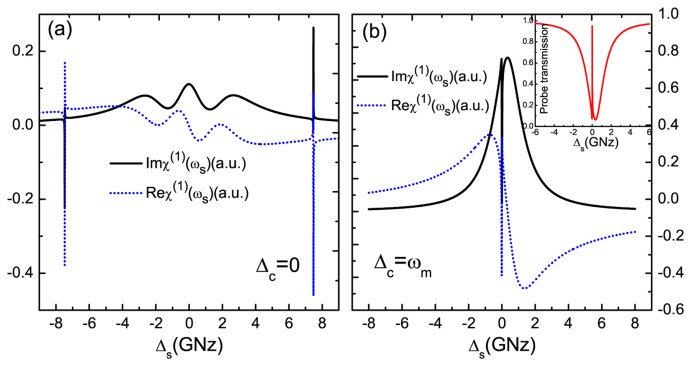
**(a)** The imaginary and real part of the linear optical susceptibility as a function of the probe-exciton detuning Δ*s* at the pump laser on-resonant with exciton frequency Δ*_c_* = 0; **(b)** the imaginary and real part of the linear optical susceptibility as a function of Δ*_s_* under off-resonant Δ*_c_* = *ω_m_*, and the inset shows the transmission of the probe laser. The parameters used are *γ_m_* = 0.83 MHz, Γ_2_ = 1 GHz, *ω_m_* = 7.477 GHz, ζ = 0.09, and $\Omega_{c}^{2}=1.0{\left(\text{GHz}\right)}^{2}$.

We consider a Z-shaped GNR of dimensions (*L, W*) = (14.1,0.7) nm (*L* and *W* indicate the length and width of the GNR resonator, respectively) composed of 424 carbon atoms [33,50] with an ambient temperature of 10 K. The model is shown in Figure 1, and the parameters of GNR resonator are shown as follows [33,50]: the fundamental vibration frequency is *ω_m_* = 7.477 GHz; the quality factor is *Q* = 9,000; the decay rate *γ_m_* = *ω_m_*/*Q*; the effective mass is *M* = 0.73 × (424*m_C_* + 140*m_H_*) = 6340 yg (*m_C_* = 1.993 yg, *m_H_* = 1.674 yg) (1 yg= 10^−27^ kg); the exciton dephasing rate Γ_2_ = 1 GHz; and the exciton-resonator coupling strength is about ζ = 0.09 [43]. In Figure 2a, we first show the imaginary part (Im*χ*^(1)^) and the real part (Re*χ*^(1)^) of linear optical susceptibility as functions of probe-exciton detuning Δ*_s_* = *ω_s_* − *ω_e_* at the detuning of the exciton frequency and the pump frequency Δ*c* = 0, which corresponds to the absorption and dispersion of the probe laser, respectively. From the curves, we find that there are two sharp peaks at both sides of the spectra that just correspond to the vibrational frequency of the GNR resonator, and the middle parts indicate the absorption and dispersion of the exciton in the GNR resonator. The physical origin of the phenomenon has been demonstrated in a coupled nanomechanical resonator system [32]. In the case of red sideband Δ*_c_* = *ω_m_*, the imaginary part and real part of linear optical susceptibility exhibit zero absorption and a positive steep slope at Δ*_s_* = 0, as shown in Figure 2b. Furthermore, at the red sideband, an analogous phenomenon of optomechanically-induced transparency [36] will appear in the system, and we term it as phonon-induced transparency (PIT) [51], as shown in the inset of Figure 2b. This is due to mechanically-induced coherent population oscillation when the pump-probe detuning equals the GNR resonator frequency [32].

**Figure 3.**
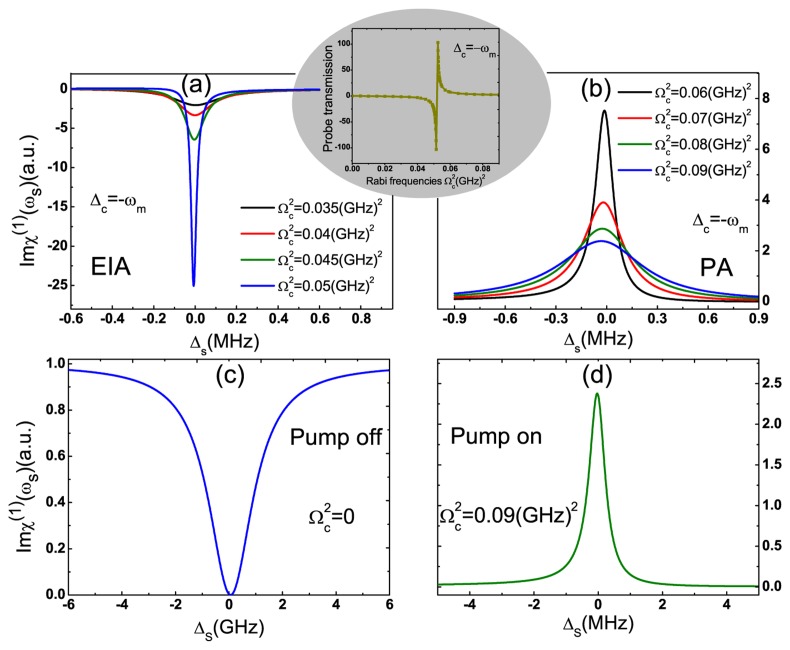
**(a,b)** The probe laser transmission as a function of Δ*_s_* with the increase of Rabi frequencies of the pump field at Δ*_c_* = −*ω_m_*. The oval inset shows the relationship between probe transmission and pump laser intensity; **(c,d)** Attenuation and amplification of the probe laser when turning off and on the pump laser, respectively. The other parameters used are the same as Figure 2.

Switching the pump-exciton detuning to the blue sideband Δ*_c_* = −*ω_m_* and increasing the pump field intensity, the probe transmission displays a deeper dip, as shown in Figure 3a. In Figure 3a, the negative transmission of the probe laser with the increase of the pump intensity is the so-called electromagnetically-induced absorption (EIA) [52]. However, with further increasing the pump laser intensity, the system switches from EIA to parametric amplification (PA), resulting in the probe laser amplification, as shown in Figure 3b, which has been demonstrated in the conventional optomechanical system [52]. The elliptical inset in Figure 3 shows that there exists a turning point among $\Omega_{c}^{2}=0.05{\left(\text{GHz}\right)}^{2}$ and $\Omega_{c}^{2}=006{\left(\text{GHz}\right)}^{2}$, which switches the probe transmission from EIA to PA. Therefore, the GNR resonator system cannot only switch the weak probe laser from off to on, but can also serve as a quantum optomechanical transistor due to the probe amplifier effect. Turning off the pump laser, the weak probe laser displays the transmission spectrum owning to the usual exciton absorption resonance, as shown in Figure 3c. However, turning on the pump laser and fixing the pump-exciton detuning Δ_c_ = −*ω_m_*, the dip switches to a transmission peak immediately, as shown in Figure 3d. This amplification comes from the quantum interference between the phonons and the beat of the two optical fields via the exciton in the GNR resonator. Due to dressing with the phonon modes, the original two levels of excitons in the GNR resonator system split into several metastable levels. When applying a strong pump laser to the system, the electrons can transit between the metastable levels, which induces the constructive interference and eventually amplifies the weak probe laser.

**Figure 4.**
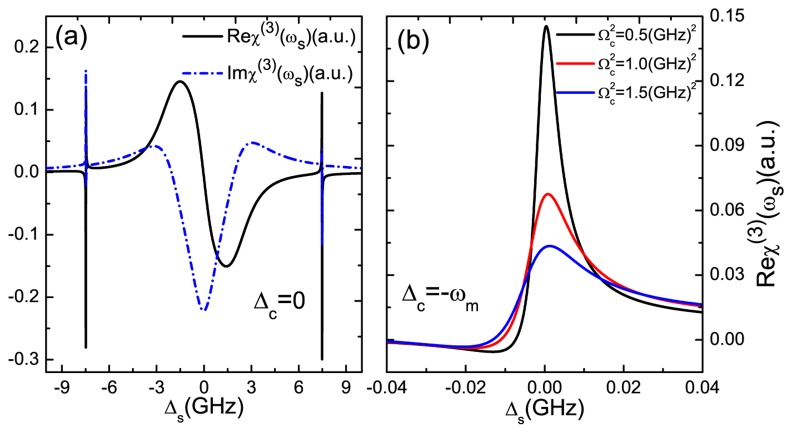
**(a)** The optical Kerr coefficient and nonlinear absorption as functions of Δ*_s_* at Δ*_c_* = 0; **(b)** The optical Kerr coefficient as a function of the detuning Δ*_s_* for several different Rabi frequencies under Δ*_c_* = −*ω_m_*. The other parameters used are the same as Figure 2.

In addition, we also demonstrate the nonlinear optical properties of the GNR resonator system. Figure 4a plots the optical Kerr coefficient Re*χ*^(3)^ (black curve) and nonlinear absorption Im*χ*^(3)^ (blue curve) as functions of probe-exciton detuning Δ*_s_* at Δ*_c_* = 0. If fixing the pump laser on-resonance with the exciton frequency in the GNR resonator and scanning the probe laser, the large enhanced optical Kerr effect can be obtained at Δ*_s_* = ±7.477 GHz. The origin of this phenomenon is the quantum interference between the vibration mode of the GNR resonator and the beat of the two optical fields via the exciton when probe-pump detuning *δ* is adjusted equal to the GNR resonator frequency [32]. Then, we adjust the pump-exciton detuning at Δ*_c_* = −*ω_m_*; the probe laser experiences different optical Kerr coefficients with different pump laser intensities, as shown in Figure 4b. By increasing the intensity of the pump laser, the optical Kerr effect will be weakened significantly. Therefore, the magnitude of the optical Kerr effect can be tuned via controlling the pump intensity, which presents a method for modifying the nonlinear optical features of GNR resonator.

**Figure 5.**
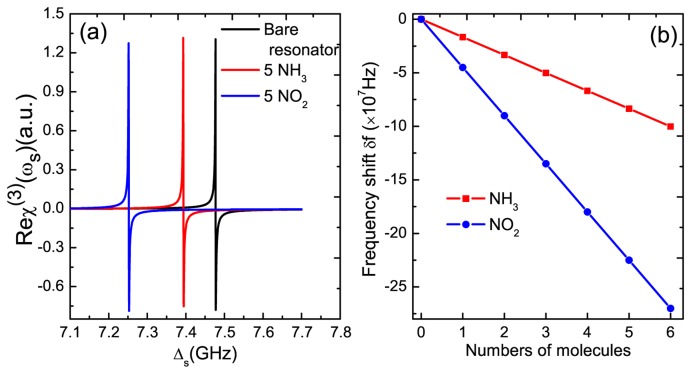
**(a)** The optical Kerr coefficient with and without landing the external molecules on the surface of the GNR resonator. The black curve shows the original resonance of the resonator; the red curve indicates that after landing five NH3 molecules, and the blue curve shows that after depositing five NO2 molecules; **(b)** The relationship between the frequency shift of the resonator and the number of deposited molecules. The other parameters used are the same as Figure 2.

On the other side, to realize the application in mass sensing is the primary goal for the research of GNR resonator systems. In order to implement mass sensing, the first step is to determine the original frequency of the GNR resonator. In Figure 4a, we find that the two sharp peaks induced by the exciton-resonator coupling in the GNR resonator locates accurately at the resonator frequency Δ*_s_* = ±*ω_m_*. This indicates a nonlinear optical scheme for measuring the frequency of the GNR resonator. The physical origin of this result is due to mechanically-induced coherent population oscillation, which makes quantum interference between the resonator and the beat of the two optical fields via the localized exciton when the probe-pump detuning is equal to the resonator frequency. Therefore, if we tune the pump beam properly and scan the probe frequency across the exciton frequency in the spectrum, we can easily obtain the accurate vibration frequency of the GNR resonator. The implementation of the nanomechanical mass sensor depends on monitoring the resonance frequency variation of a nanomechanical resonator when additional mass is adsorbed onto its surface. Once additional mass is deposited on the surface of the resonator, the new frequency of the resonator can be measured, then additional mass can be determined straightly via Equation (10). Here, we propose a nonlinear optical mass sensing via using the Kerr coefficient based on the GNR resonator system. In Figure 5a, we first deposit five NH_3_ molecules on the GNR resonator and measure the new frequency of the resonator. There is a frequency shift *δf*_1_ = 83.5 MHz (see the red curve) compared with the bare resonator without depositing any molecules (see the black curve) onto it. Similarly, five NO_2_ molecules can also be estimated via the frequency shift *δf*_2_ = 225.2 MHz in the probe nonlinear Kerr spectroscopy (see the blue curve). Figure 5b shows the linear relationship between the frequency shifts and the number of deposited molecules. The negative slope gives the mass sensitivity of the resonator. In this way, once the frequency of the resonator is determined, the mass of the resonator can also be obtained accurately via the slope.

To evaluate the sensitivity of the resonator in mass sensing, one significant parameter of mass responsivity ℜ = *ω_m_*/(2*M*) is introduced. Obviously, the lower mass, higher vibration frequency and higher quality factor of the resonator may improve the sensitivity of the sensor effectively. For a practical GNR structure, it will exhibit a wide range of variations in parameters, such as mass, quality factor, exciton-resonator coupling strength, dephasing rate, *etc.* In the present work, we only consider the parametric values, as shown in the above figures. The change of the size in the GNR resonator will cause the variations in the mass, the resonance frequency and the quality factors. These size-dependent parameters will influence the sensing performance and the sensitivity. The exciton-resonator coupling strength plays a key role in the MoS_2_ resonator system. If the coupling strength is much larger than ζ = 0.09, there will be a remarkable enhancement of the optical Kerr effect, which will benefit for the performance of the sensor. Further, the dephasing rate of the exciton and the lifetime of the resonator will also affect the performance of the sensor, which have been discussed in detail in the carbon nanotube resonator and a hybrid nanocrystal coupled to a nanomechanical resonator [32]. Experiments carried out in a dilution refrigerator are the drawback of our proposed mass sensing scheme as compared with room temperature mass spectrometry. If the system works as mass sensors at room temperature, the mass sensitivity will be decreased. However, our nonlinear optical mass sensing could be used to monitor nonliving objects, such as N_2_ molecules and Cr atoms.

In the implementation of mass sensing, both intrinsic and extrinsic noise sources will affect the sensitivity of the sensing devices [53,54]. Thermomechanical noise is one dominant intrinsic noise that will affect the ultimate sensitivity, and implementing the mass sensing in a cryogenic environment can reduce such noise. a mass sensor operated in a linear domain is essential for the use of low-noise optical and electronic detectors when measuring the output signal, and detection noise may be inappreciable in this situation. Furthermore, the mass resolution limits imposed by the intrinsic thermomechanical noise can also be given via the expression $\delta f\approx 10^{-DR/20}\sqrt{BW\times 2\pi \omega_{m}/Q}$, where DR is the dynamic range in units of decibels and BW is the measurement bandwidth, which further reveals that the small effective mass and high quality factor *Q* of the resonator are crucial for improving the mass sensitivity [48]. However, for many practical applications relying on sophisticated readout equipment, the detection noise as an extrinsic noise source is significant, which will not only obstruct precise sensing, but also will generate the limit of detection of the sensor [53]. Theoretical and experimental investigations have shown that nonlinear behaviors can enhance the sensitivity of the mass sensing [55]. The mass sensor in the nonlinear regime induces large oscillation amplitudes and large output signals without simultaneously amplifying the noise. These benefit counteracting the influence of detection noise and improve the signal-to-noise ratio. Under this condition, compared with the mass sensing in the linear optical region, the use of the nonlinear optical spectrum may overcome the detection noise and offer better performance over the linear optical spectrum where detection noise is the main factor that determines sensitivity [53,54].

We have assumed that the deposited molecules distribute uniformly along the surface of the resonator. Actually, the frequency shift depends on both the deposited mass and its position of adsorption on the surface of the nanomechanical resonator. For the GNR resonator system, the maximum shift is obtained at the center for the fundamental mode of vibration, while the minimal shifts are induced for the adsorption near the edge points. This statistical distribution of frequency shifts has been investigated by building the histogram of event probability *versus* frequency shift for small ensembles of sequential single molecule or single nanoparticle adsorption events [28].

In our all-optical, nonlinear mass sensor, the particle mass to be measured does not need ionization, and its mass can be directly measured from the nonlinear Kerr spectroscopy conveniently. Our optical mass sensing scheme can also effectively avoid the drawbacks that cause the heating effects based on the electrical measurement, and the nonlinear optical mass sensing may be immune to the detection noises. Besides, the pump-probe scheme generates a beat wave to drive the mechanical resonator, which allows both the high and low frequency of the mechanical resonator. Due to the excellent properties of graphene-based nanoresonator, the nonlinear optical mass sensing proposed here can even reach the detection of single atoms.

## 4. Conclusion

We have proposed a theoretical model based on a Z-shaped GNR resonator with the optical pump-probe scheme to investigate its coherent optical properties. The phenomenon of phonon-induced transparency, electromagnetically-induced absorption and parametric amplification are demonstrated by the exciton-resonator coupling in the system, which may suggest a quantum optical transistor. By switching the pump-exciton detuning and manipulating the intensity of the pump laser, the optical Kerr effect of the system can be tuned. We further put forward a nonlinear optical mass sensor based on a Z-shaped GNR resonator via the nonlinear optical properties. This nonlinear optical mass sensor may be applied to environmental monitoring and chemical detection and even the measurement of a single atomic mass, due to the excellent properties of the GNR resonator system. Finally, we anticipate that our nonlinear optical detection scheme can be implemented in current experiments.
